# Missing the boat: odds for the patients who leave ED without being seen

**DOI:** 10.1186/1471-227X-13-1

**Published:** 2013-01-16

**Authors:** Jabeen Fayyaz, Munawar Khursheed, Mohammed Umer Mir, Amber Mehmood

**Affiliations:** 1 Department of Emergency Medicine, Aga Khan University Hospital, Karachi, Pakistan

## Abstract

**Background:**

A patient left without being seen is a well-recognized indicator of Emergency Department overcrowding. The aim of this study was to define the characteristics of LWBS patients, their rates and associated factors from a tertiary care hospital of Pakistan.

**Methods:**

A retrospective patient record review was undertaken. All patients presenting to the Aga Khan University Hospital, Karachi, between April and December of the year 2010, were included in the study. Information was collected on age, sex, presenting complaints, ED capacity, month, time, shift, day of the week, and waiting times in the ED. A basic descriptive analysis was made and the rates of LWBS patients were determined among the patient subgroups. Logistic regression analysis was used to assess the risk factors associated with a patient not being seen in the ED.

**Results:**

A total of 38,762 patients visited ED during the study period. Among them 5,086 (13%) patients left without being seen. Percentage of leaving was highest in the night shift (20%). The percentage was twice as high when the ED was on diversion (19.8%) compared to regular periods of operation (9.8%). Mean waiting time before leaving the ED in pediatric patients was 154 minutes while for adults it was 171 minutes. More than 32% of patients had waited for more than 180 minutes before they left without being seen, compared to the patients who were seen in ED. Important predictors for LWBS included; Triage category P4 i.e. walk –in-patients had an OR of 13.62(8.72-21.3), Diversion status, OR 1.49(1.26-1.76), night shift , OR 2.44(1.95-3.05) and Pediatric age, OR 0.57(0.48-0.66).

**Conclusions:**

Our study elucidates the LWBS population characteristics and identifies the risk factors for this phenomenon. Targeted interventions should be planned and implemented to decrease the waiting time and alternate services should be provided for high-risk patients (for LWBS) to minimize their number.

## Background

Emergency Departments (ED) not only provide care to patients with critical and life threatening emergencies, but also look after round the clock to those who have acute yet stable medical illnesses [1-3]. The resultant ED overcrowding which was first described twenty years ago, has now become a well-established barrier in access to health care [4-6]. The problem is exacerbated in low income countries by utilization of ED as a primary access point to the healthcare especially on weekends and after hours for less urgent conditions [1]. However, the balance is now tilting towards high acuity patients, ED boarding of admitted patients, and hospital occupancy as a cause of ED overcrowding rather than influx of non-urgent patients [4,5,7]. ED overcrowding not only reduces patient satisfaction but it also increases the number of patients that leave without being seen by a physician (LWBS) [3,7]. Large number of these patients may not find appropriate care elsewhere and therefore a critical treatment opportunity is missed by the health system. The percentage of LWBS patients has been recognized as a proxy indicator of ED performance and overcrowding [8,9].

A number of studies from high income countries with well-established primary health care system have reported a variable number of LWBS which ranges between <1% to 20% of all ED visits [10-14]. It has been suggested that patients who LWBS are at an increased risk of morbidity and mortality; however, a more recent administrative follow-up demonstrated these patients are at a lower risk of hospitalization and death than triage-matched controls [15-19]. Several factors have been found as being associated with LWBS, such as low acuity illness, young age, and male sex and prolonged waiting time [20-24]. Additionally, the triage time, previous ED visits, seasonal variation, access to primary care, diversion status and ED overcrowding also have significant impact on LWBS [10,25-29]. A literature review of local published research from Pakistan showed no study documenting the characteristics of patients who leave ED without being seen by a physician in this region.

Emergency Medicine as a specialty is still in its infancy in Pakistan [30-32]. Our department was the first one to be established back in 2008. Over the years, we have observed an increase in patient volume as well as acuity. The ED had expanded to 46 beds but the hospital beds remained the same which brought in the issues of overcrowding, left without being seen patients and ED through put issues. Therefore, this study is aimed at defining the LWBS population in a tertiary care hospital while determining percentages and factors associated with LWBS as we do not know the characteristics of our patients who are leaving. This baseline information will be critical in developing evidence based interventions aimed at improving the health care management of such patients and consequently reducing the morbidity and mortality resulting from leaving.

## Methods

### Setting

This study was conducted at the Emergency Department of the Aga Khan University Hospital (AKUH) Karachi, Pakistan. AKUH is a 600-bedded, private tertiary care hospital in Karachi with an annual ED census of approximately 50,000 patients and an admission rate of 37%. The emergency department of AKUH is the first one established in the country, and the largest ED in Pakistan providing emergency care of international standards.

The emergency department of AKUH is the first established department at Pakistan. It has 46 patient-care beds with well designated pediatric, critical care areas and non-critical areas. An eight-bedded observation unit is also functioning where patients are kept for 24 hours. Two Fast track clinics for walk in patients provide service 24/7. AKUH-ED is the only department in Pakistan where standard triage is being followed (Additional file 1). It has a separate well defined triage area. It follows 4 levels of triage and categorizes patients from level I-level IV. We also have a written triage policy approved by the hospital. Triage staff has been given training for Triaging. A nurse initially triages patients by following the triage categories. The nurse assigns beds to the patients or sends them to the waiting area in case the ED capacity is exhausted (Additional file 2). At the triage desk, a triage team is present 24/ 7 comprising of a trained nurse, nursing assistant and a triage care coordinator. Triage care coordinator is a senior experienced nurse who supervises the whole functioning of triage. In case of any quarry, the triage nurse could seek help from an on-call physician.

The triage information is recorded in an electronic computerized based system called ERMS (Emergency Room Management system) (Additional file 3). It is software that was developed by the information technology department of Aga Khan University Hospital (AKUH). It has two components, Triage assessment and waiting list.

Every staff working in ED has been given a login ID and password for logging in. After logging, in the windows shows two options: triage assessment and waiting patient work list. For triage assessment, the triage staff would click on the “TRIAGE ASSESSMENT” icon which opens up a new window asking for the patient details like medical record number, vitals, presenting complaints etc. After entry, this information is saved in the computer and can be retrieved later for analysis. This software also helps the staff in identifying abnormal vital signs like heart rate, blood pressure and oxygen saturation according to the age of the patients by blinking vitals in red. This way, it assists the staff in triaging the patients correctly. This study had been approved by the Ethical Review Committee (ERC) of Aga Khan University.

After filling all these information, the patient triage category triage assessment number and the bed is assigned if available. In case of non-availability of bed and the patient is not life threatening or critical, then the patient is transferred to the waiting area and this information can be reviewed by the staff later on by clicking “waiting patient list”. In this way the staff completes the triage process for patients.

ED staff can review bed statistics any time by using the same software. When a bed becomes available in the ED or the defined waiting time is completed, the patient is called again for reassessment or allocation of bed. At this point they are asked to go to the registration desk and were registered with their medical record number for patients who had visited the AKUH previously as well. If that patient is visiting for the first time than a new medical record number is allocated.

When patient is assigned a bed in the ED, after waiting than this time is measured as waiting time before getting a bed. When a bed is made available than the name of the patient is called for three times by triage staff at 2 minutes interval and if a patient does not reply , they are labeled as “left without being seen” and that time is noted as their waiting time.

Return visits are recorded if the patient after leaving the emergency department comes back within 48 hours of visit. The return visits are usually tracked down through the medical record number.

### Data collection

All patients who were triaged in the Emergency Department of AKUH from April 1, 2010 to December 31, 2010, are included in the study. This time period was chosen to ensure consistency of results as we implemented a defined triage policy so to exclude any bias time period from Jan –March. We used an electronic ED record system to extract clinical data of all patients who were triaged in the AKUH-ED. Information on age, sex, complaints, and triage category, time of arrival, day of arrival, time and shift of the day when patient left the ED was recorded. Information was available on 38,762 patients for inclusion.

Four-level triage scale (P1 to P4) is used in the AKUH ED and was used for the purpose of this study. Patients with life threatening conditions are labeled P1, those in a critical state are labeled P2, P3 are patients who require urgent medical care, and P4 are walk-in stable patients. When all the ED beds are occupied, non-critical patients are usually asked to wait till a bed is available for them.

In this study, diversion means a situation in which the ED continues to accept critical patients despite of full occupancy but less critical patients are diverted to other healthcare facilities. This diversion status is reviewed every four hours.

### Statistical analysis

Data of patients, who were treated in ED and those with LWBS visit, was compiled and analyzed. Proportions were calculated for both groups and significant differences were assessed using the Chi-square test. Percentages of LWBS visits along with their 95% CIs were then calculated for all categories.

We used logistic regression to assess association of patient characteristics with LWBS visits at the univariate and multivariable level. For the logistic regression, LWBS visit status was taken as the outcome and its relationships with independent variables including sex of patient, age, and triage level, diversion status of ED, month and day and time of presentation. Waiting times in the ED before leaving were not included in the model due to uncertainty in their accuracy (exact value was difficult to ascertain as LWBS status was only identified when the staff called for the patient. The patient could have left any time after the initial triage). All variables were included at the univariate level and a p-value of 0.25 was considered the cut-off for inclusion in the multivariable model. The enter method was used to derive the final regression model. Unadjusted and adjusted odds ratios (ORs) are presented in the results. SPSS version 19 was used to analyze the data. An exemption of ethical approval was given by the Ethical Review Committee on 13th May, 2011 at Aga Khan University.

## Results

A total of 38,762 patients were triaged from April to December 2010 at AKU - ED and were included in our study; 13866(35.33%) were admitted. Total 5,086 patients left the ED without getting medical care during this period, giving an overall rate of over 13% over 9 month period.

**Figure 1:**
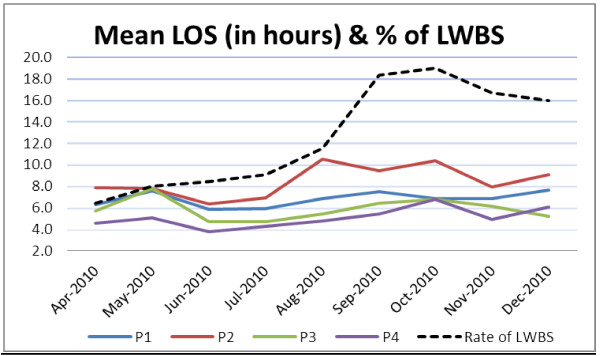
Relationship of length of stay, triage category and LWBS patients.

There were significant differences between those patients receiving medical care and those who left before treatment (Table 1). Percentage of LWBS visits for females was slightly higher than males but the relationship between sex of patient and LWBS visits was not significant in the multivariable regression model (Tables 2 &3). For triage level assignments, proportion of patients in P3 categories was highest (15.7%) who left without being seen, which means that they were seven times more likely to be in a LWBS visit compared to P1 & P2 patients. The length of stay and the percentage of patients leaving were also increased with increased number of P1 and P2 patients (Figure 1). LWBS percentages seem to vary with time of the day and were more than 20% in the night shift (11 pm to 7 am) compared to about 4% in the morning shift (7am to 3pm). This finding was found to persist in the regression analysis, which revealed a 2.6 times higher odds of an LWBS visit if the patient presented to the ED in the night shift compared to the morning hours. Another important predictor of LWBS visit is the diversion status of the ED at the time of presentation. Patients visiting during the ED diversion hours are 1.5 times more likely to have a LWBS visit than when diversion status is off (19.8% vs. 9.8% during off-diversion). Sex and day of the week on which the patient presented showed an association with LWBS visits at the univariate level, but this relationship was not found after adjustment with other factors in the multivariable model (Tables 2 &3). Percentage of LWBS is more in female (13.75) patients as compared to male (12.58).

**Table 1:** Basic demographic characteristics of patients

	**Seen by physician** (**N**=**33676**)	**Left without being seen** (**N**=**5086**)	**P**- **value**
	**n (%)**	**n (%)**	
**Sex**			
Male	18232 (54.1)	2634 (51.8)	0.031
Female	15444 (45.9)	2452 (48.2)	
**Age**			
<14yrs	9816 (29.1)	933 (18.3)	<0.001
14-20yrs	1824 (5.4)	311 (6.1)	
20-40yrs	8812 (26.2)	1791 (35.2)	
40-60yrs	7091 (21.1)	1205 (23.7)	
60-80yrs	5319 (15.8)	722 (14.2)	
80yrs and above	814 (2.4)	125 (2.5)	
	**Triage**			
P1 & P2	6850 (20.3)	90 (1.8)	<0.001	
P3	23564(70.0)	4409(86.7)		
P4	3262 (9.7)	587(11.5)		
**Diversion Status**				
Off Diversion	23441 (69.6)	2558 (50.3)	<0.001	
On Diversion	10235 (30.4)	2528 (49.7)		
**Months**				
April	3356 (10.0)	231 (4.5)	<0.001	
May	3656 (10.9)	319 (6.3)		
	June	3489 (10.4)	320 (6.3)	
July	3720 (11.0)	370 (7.3)		
August	3707 (11.0)	483 (9.5)		
September	4085 (12.1)	916 (18.0)		
October	4253 (12.6)	996 (19.6)		
November	3918 (11.6)	786 (15.5)		
December	3492 (10.4)	665 (13.1)		
**Weekdays**				
Saturday	5057 (15.0)	644 (12.7)	<0.001	
Sunday	5203 (15.4)	955 (18.8)		
Monday	4768 (14.2)	753 (14.8)		
Tuesday	4655 (13.8)	693 (13.6)		
Wednesday	4584 (13.6)	661 (13.0)		
Thursday	4588 (13.6)	711 (14.0)		
Friday	4821 (14.3)	669 (13.2)		
**Waiting time** (**min**)				
0-30	24035 (71.4)	502 (9.9)	<0.001	
31-60	2485 (7.4)	597 (11.7)		
61-120	2832 (8.4)	1327 (26.1)		
121-180	1502 (4.5)	998 (19.6)		
>180	2822 (8.4)	1662 (32.7)		
**Shift**				
7am - 3pm	11442 (34.0)	514 (10.1)	<0.001	
3pm - 11 pm	12727 (37.8)	2145 (42.2)		
11pm-7am	9507 (28.2)	2427 (47.7)		

**Table 2:** Percentages of LWBS in patient groups

	**N**	**n**	**Percentage of LWBS**	**95**% **CI**
**Sex**				
Male	20940	2634	12.58	(12.14, 13.03)
Female	17838	2452	13.75	(13.25, 14.26)
**Age**				
< 14 yrs	11015	933	8.47	(7.95, 8.99)
14-<20yrs	2119	311	14.67	(13.23, 16.25)
20-<40yrs	10389	1791	17.24	(16.53, 17.98)
40-<60yrs	8235	1205	14.63	(13.89, 15.41)
60-<80yrs	6082	722	11.87	(11.08, 12.71)
80yrs & above	939	125	13.3	(11.29, 15.64)
**Triage**				
P1 & P2	6942	90	1.3	(1.06, 1.59)
P3	27984	4409	15.76	(15.33, 16.19)
P4	3852	587	15.24	(14.14, 16.41)
**Diversion Status**				
Off Diversion	26031	2558	9.83	(9.47, 10.19)
On Diversion	12747	2528	19.83	(19.15, 20.53)
**Months**				
April	3590	231	6.43	(5.68, 7.28)
May	3976	319	8.02	(7.22, 8.91)
June	3810	320	8.4	(7.56, 9.32)
July	4091	370	9.04	(8.2, 9.96)
August	4191	483	11.52	(10.59, 12.53)
September	5002	916	18.31	(17.27, 19.41)
October	5251	996	18.97	(17.93, 20.05)
November	4706	786	16.7	(15.66, 17.79)
December	4158	665	15.99	(14.91, 17.14)
**Weekdays**				
Saturday	5704	644	11.29	(10.49, 12.14)
Sunday	6160	955	15.5	(14.62, 16.43)
Monday	5524	753	13.63	(12.75, 14.56)
Tuesday	5350	693	12.95	(12.08, 13.88)
Wednesday	5247	661	12.6	(11.73, 13.52)
Thursday	5301	711	13.41	(12.52, 14.36)
Friday	5492	669	12.18	(11.34, 13.07)
**Waiting time** (**Min**)				
0-30 min.	24353	502	2.06	(1.89, 2.25)
31-60 min.	3097	597	19.28	(17.93, 20.7)
> 61 min.	11327	3987	35.20	(34.32, 36.08)
**Shift**				
7am - 3pm	11885	514	4.32	(3.97, 4.71)
3pm - 11pm	14891	2145	14.4	(13.85, 14.98)
11pm - 7am	12002	2427	20.22	(19.51, 20.95)

**Table 3:** Patient characteristics of ED visits by Whether or not the patient left without being seen

	**LWBS** -**ve** (%)	**LWBS** +**ve** (%)	**Unadjusted OR** (**95**% **CI**)	**Adjusted OR** (**95**% **CI**)
**Triage Category**				
**P1** &**P2**	20.34	1.77	Ref.	Ref.
**P3**	69.97	86.69	14.24 (11.54-17.58)	13.62 (8.72-21.3)
**P4**	9.69	11.54	13.69 (10.93-17.15)	13.14 (8.04-21.49)
**Diversion Status**				
**Off**	69.61	50.26	Ref.	Ref.
**On**	30.39	49.74	2.26 (2.13-2.42)	1.49 (1.26-1.76)
**Age category**				
**Adult**	29.15	18.34	Ref.	Ref.
**Pediatric**	70.85	81.66	0.55 (0.49-0.61)	0.57 (0.48-0.66)
**Shifts of Day**				
**7am** - **3pm**	34	10.1	Ref.	Ref.
**3 pm** - **11pm**	37.8	42.2	3.75 (3.39-4.15)	3.47 (2.83-4.26)
**11pm** - **7am**	28.2	47.7	5.60 (5.15-6.28)	2.44 (1.95-3.05)
**Months**				
**April**	9.97	4.54	Ref.	Ref.
**May**	10.86	6.27	1.26 (1.06-1.51)	0.92 (0.66-1.29 )
**June**	10.36	6.29	1.33 (1.12-1.59)	0.747 (0.526-1.06)
**July**	11.05	7.27	1.44 (1.22-1.72)	0.73 (0.52-1.04)
**August**	11.01	9.5	1.89 (1.61-2.23)	0.96 (0.69-1.32)
**September**	12.13	18.01	3.26 (2.80-3.79)	1.79 (1.34-2.4)
**October**	12.63	19.58	3.40 (2.93-3.96)	2.21 (1.57-3.11)
**November**	11.63	15.45	2.92 (2.50-3.40)	1.42 (1.04-1.94)
**December**	10.37	13.08	2.77 (2.37-3.24)	1.27 (0.9-1.78)

We also observed a difference in percentage of LWBS over the total study duration, being lowest in April (6.4%) and highest in the September to October period (up to 19%) (Table 2). This pattern was consistent in the regression model which showed higher odds of LWBS visits in October (marginally significant), November and December (Table 3).

**Figure 2:**
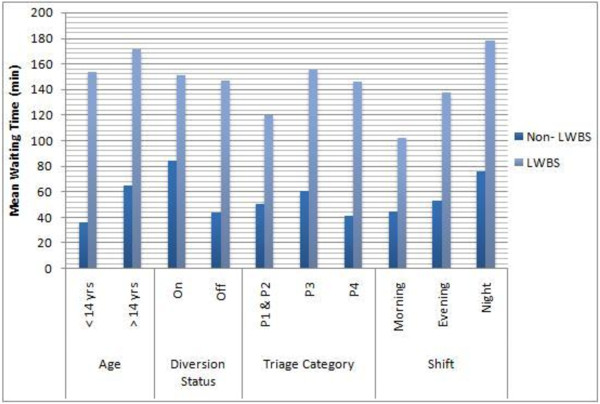
Relationship between Age, Diversion Status, shift of day and LWBS with respect to waiting time.

Median waiting time for pediatric patients was 154 minutes and 171 minutes for adults who left. Patients with a waiting time of over 180 minutes had 26 times higher odds of leaving compared to those who waited for less than 30 minutes. This relationship is consistent in the adjusted multivariable model (Table 3). Relationship of wait time with age, triage category diversion status and shift of the day had been shown in Figure 2.

**Figure 3:**
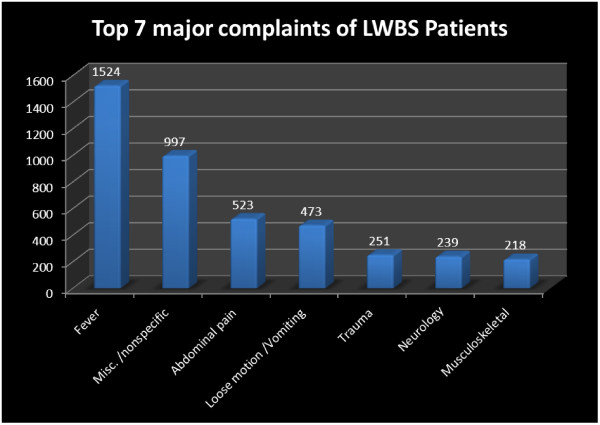
Top Seven Complaints of patients who left without being seen.

The top seven presenting complaints of patients with LWBS visits are shown in Figure 3. Most of the cases were of fever, non-specific complaints, abdominal pain, and vomiting/ diarrhea. Co-morbid was identified in 12.6% of patients with LWBS visits. Total patients who returned to hospital within 48 hours were 181 in LWBS group vs. 251 among the patients who were discharge; which means overall 3.6% of the LWBS patients vs. 1.11% of discharged patients needed to revisit in ED for medical care. Among the LWBS, 77 (1.5%) and 6(0.26%) in the discharge group required admission to the inpatient units (Table 4).

**Table 4:** Characteristics of patients with return visits by months

**Visit month year**	**Total patient**	**Left patient**	**Rate of return visitsin LWBS n** (%)	**Rate of admission inLWBS n** (%)	**Discharge patients**	**Rate of return inD**/**C n** (%)	**Rate of admission inD**/**C n** (%)
**Apr**-**2010**	3356	231	8(3.6)	1(0.4)	2176	13(0.59)	1(0.04)
**May**-**2010**	3656	319	10(3.1)	3(0.9)	2335	14(0.6)	0
**Jun**-**2010**	3489	320	16(5.0)	4(1.3)	2322	15(0.64)	1(0.04)
**Jul**-**2010**	3720	370	11(3.0)	3(0.8)	2556	23(0.9)	0
**Aug**-**2010**	3707	483	17(3.5)	6(1.2)	2379	35(1.47)	0
**Sep**-**2010**	4085	916	48(5.3)	20(2.2)	2979	45(1.51)	1(0.03)
**Oct**-**2010**	4253	996	36(3.6)	15(1.5)	2933	43(1.46)	3(0.10)
**Nov**-**2010**	3918	786	19(2.4)	8(1.0)	2732	42(1.53)	0
**Dec**-**2010**	3492	665	17(2.6)	12(1.8)	2188	21(0.96)	0
**Total**	**33676**	**5086**	**181**(**3**.**6**)	**77**(**1**.**5**)	**2260**	**251**(**1**.**11**)	**6**(**0**.**26**)

## Discussion

To the best of our knowledge, this is the first study to describe the characteristics of Pakistani patients who left emergency department without being seen by a physician from a tertiary care hospital. We have described the relationship of LWBS with age, triage category, day of week, and shift of day, diversion status and waiting time. We found that in this study LWBS were 13% which is comparable to other international data (1.0 – 15%) but higher than the benchmark set by USA (1.7%) [16,17,33].

Although the sex of those who LWBS does not have significant effect in a multiple regression model, it appears that age of a patient had a profound impact (Table 3). The odds of leaving for male patient who is 20–40 years of age is 17 times more than a patient at extremes of age, regardless of severity of illness. Children were found to be at a lower risk of being left, this may show increased sensitivity and nonspecific sign and symptoms towards extremes of age that gives them priority over other age groups [15,23,34]. Proportion of LWBS are higher in females contrary to international data may be because in our community structure females have the responsibility of taking care of all the household things as well their health is not given as much priority because of existing inequities in our communities [35,36].

It was observed that higher proportions of low acuity (98.2%) patients with less severe illnesses like fever, upper respiratory tract infection (URTI), acute gastroenteritis were leaving. Research has shown that LWBS and acuity has a dose- response relationship; with 15.2% of non-urgent patients leaving as compared to 0.1% of critical patients [10,13,37]. The fact that most of those patients who left although had low acuity illnesses yet they required some work up or treatment e.g. abdominal pain or diarrhea with dehydration, highlights the importance of accessibility of urgent care settings or short stay units. This could be assessed by the percentage of subsequent return visit in the ED after leaving. The return visits in our study are found to be 3.6% much higher than the internationally reported numbers (1.2%) in a USA study with 1.5% requiring hospital admission subsequently [38].

A high number of walk-in patients such as those with fever or URTI utilize the ED mostly in after hours, and usually spend a long time in waiting because of their relatively stable condition. This fact also emphasizes the need for creating structures such as fast track Clinics or urgent care centers that cater the high influx of patients with seasonal illnesses who need not be referred to a tertiary care hospital for treatment and a separate patient care area for elderly patients [39-42].

From the results, it appears that patients who are asked to wait for a longer time period are also more likely to leave than those who are assigned bed within a relatively shorter time span. The odds for leaving in this study are 0.2% with every10 minutes increase in waiting time. Although the accuracy of waiting time duration is uncertain, percentages have been described in other studies [17,22,27,29,37,43-45]. Probable reasons could be either patients got tired of waiting, seek advice in another healthcare facility or they felt better and left [15,20,46,47].

The contributory factors for LWBS are overcrowding due to high patient influx and boarded patients in ED, lack of awareness among general population regarding ED utilization as well as inefficient primary health care facilities [20,39,48-52]. This crowding result into prolong waiting hours and ultimately increased rate of LWBS. In our institution because of lack of availability of inpatient beds in high acuity areas these patients who are either critically ill or intubated have to stay in the ED at times for more than 24–48 hours before their final disposition. The situation further worsen when more and more critical patients continue to land in the emergency department with limited resources like nursing staff and beds available. It is a proven phenomenon that when ED was crowded and on diversion there was 2.26 times risk of leaving the ED. Similar results have been reported by TL Viet and K V Rhodes that ED crowding increases the LWBS rate [21].

Increased percentages of LWBS during weekend or night shift and seasonal variations gives insight into epidemics such as dengue fever, inadequate outpatient services on the weekends and after hour’s utilization of ED services for minor illnesses [16,21,51,53-56]. Our data had demonstrated a sudden increase in LWBS patients in the third quarter that coincide with the dengue epidemic of 2010 in Pakistan [57,58]. A strong seasonal variation with highest LWBS (up to 70%) in winters is also found in other studies [45].

There are certain limitations of this study. First data were collected retrospectively. Secondly the study was conducted in a single tertiary care private hospital therefore results may not be generalized. Our department is the first in Pakistan to practice a defined triage system which started recently. Very little is known about the reliability and validity of the triage at our institute. This is the first ever analyzed data from AKUH-ED. Follow up studies are needed to address this issue in detail.

The cross sectional design of study did not enable us to follow the clinical outcome of LWBS patients in detail. Additional studies are required to determine subsequent morbidity and mortality as well as other hospital factors affecting the percentage of LWBS. As all the patients are not the registered patients at AKUH, so the return visit of all the patients who had been triaged cannot be traced for any adverse outcome. This was the first reported data so we haven’t studied the different age group characteristics separately. Subsequent studies on pediatric, adult and geriatric patients are needed to further elaborate their characteristics and factors affecting their decision of leaving.

## Conclusions

In this study, we found certain factors were strongly associated with LWBS such as age, low triage acuity, weekend, and night shift presentation and prolonged waiting times. ED Diversion was also associated with higher odd of leaving. This is only a single center data from a private tertiary care hospital and figures could be different in other public or private settings.

Strategies should be designed to shorten the waiting time and additional outpatient facilities such as fast track clinics to reduce the burden of these patients from ED and avoid possible bad outcome in this population who miss the opportunity of health care provision due to weak primary care facilities.

## Abbreviations

ED: Emergency Department; LWBS: Left without Being Seen; OR: Odd Ratio; CI: Confidence Interval; AKUH: Aga Khan University Hospital; P1: Priority Level 1; P2: Priority Level 2; P3: Priority Level 3; P4: Priority Level 4; URTI: Upper Respiratory Tract Infection; HTN: Hypertension.

## Competing interests

The authors declare that they have no competing interests.

## Authors’ contributions

JF and MK contributed equally to the work. MUM participated in the design and data analysis. JF and MK made the draft. AM reviewed the manuscript and gave the final approval. All authors read and approved the final manuscript.

## Pre-publication history

The pre-publication history for this paper can be accessed here:

http://www.biomedcentral.com/1471-227X/13/1/prepub

## Supplementary Material

Additional file 1 Patient Flow in ED through Triage Desk: It describes the flow of patients in the emergency department of AKUH at AKUH -ED. Click here for file

Additional file 2 Triage Categorization. Click here for file

Additional file 3 Electronic Record Management System functionality. Click here for file
