# Supplementary figures and images for: Missing the boat: odds for the patients who leave ED without being seen
Source: BMC Emerg Med. 2013 Jan 16;13:1. doi: 10.1186/1471-227X-13-1 (PMC3571890; doi:10.1186/1471-227X-13-1)

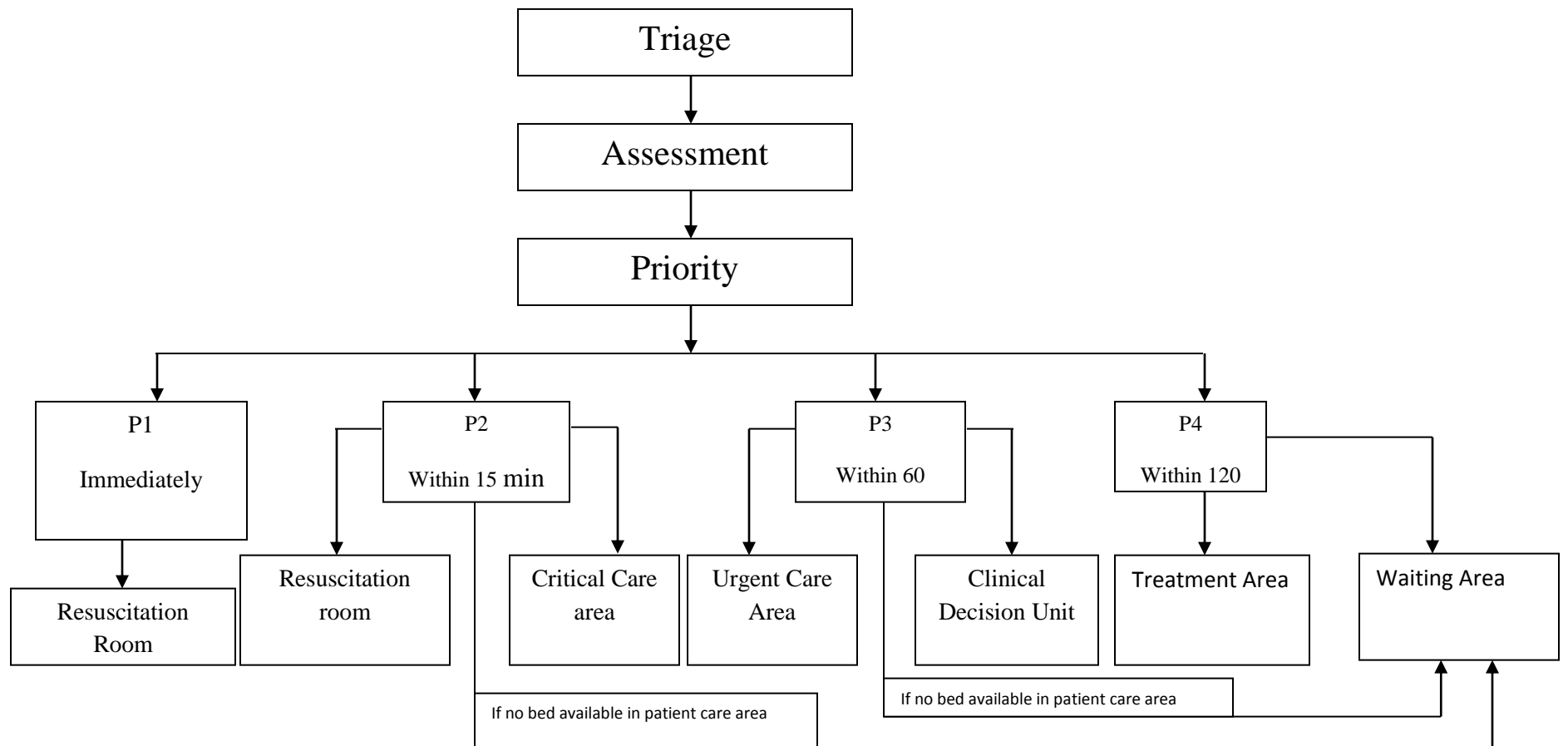

Supplement: Additional file 1 — Patient Flow in ED through Triage Desk: It describes the flow of patients in the emergency department of AKUH at AKUH -ED. [file 1471-227X-13-1-S1.pdf]
