# Supplementary material for: Missing the boat: odds for the patients who leave ED without being seen
Source: BMC Emerg Med. 2013 Jan 16;13:1. doi: 10.1186/1471-227X-13-1 (PMC3571890; doi:10.1186/1471-227X-13-1)
Supplement: Additional file 2 — Triage Categorization. [file 1471-227X-13-1-S2.pdf]

TRIAGE PRIORITY	MAXIMUM WAITING TIME STANDARD	CLINICALS SCENARIOS
**P-1**	**Immediately**	Patients with compromised ABC, severe bleeding, decreased level of consciousness, severe medical problems, poisoning with altered mental status, burn > 15%, Acute Airway Obstruction, drug overdose, Ongoing Seizures, trauma patients those fulfilling trauma activation criteria
**P-2**	**With in 15 minutes**	Cardiac chest pain, GCS<9,Open fracture with massive bleeding, fever >40 c , especially children & elderly, sever dehydration, SOB but able to give history, acute abdominal pain with LMP 6 weeks or Ectopic pregnancy, Pre-eclampcia, Acute Psychosis, History of Seizures, vitally unstable patients.
**P-3**	**With in 60 minutes**	Non cardiac chest pain (history and –ve EKG), SOB with no history of Asthma & can sit comfortably, poisoning with GCS 15 acute abdominal pain. Pain (not Ectopic), Depression, Eye trauma ((no threat to vision), Diarrhea with mild dehydration. Musculoskeletal trauma
**P-4**	**With in 2 hours**	Viral syndromes, respiratory infections, suture removal, mild UTI, nausea vomiting & diarrhea without dehydration, cough with underlying signs of COPD or asthma. 1st & 2nd Degree Burns of < 5%, children with fever < 39 C , minor abrasions & sprains , Insect bits without any systmatic symptoms, patients only for Drug Administration
