# Supplementary material for: Missing the boat: odds for the patients who leave ED without being seen
Source: BMC Emerg Med. 2013 Jan 16;13:1. doi: 10.1186/1471-227X-13-1 (PMC3571890; doi:10.1186/1471-227X-13-1)
Supplement: Additional file 3 — Electronic Record Management System functionality. [file 1471-227X-13-1-S3.pdf]

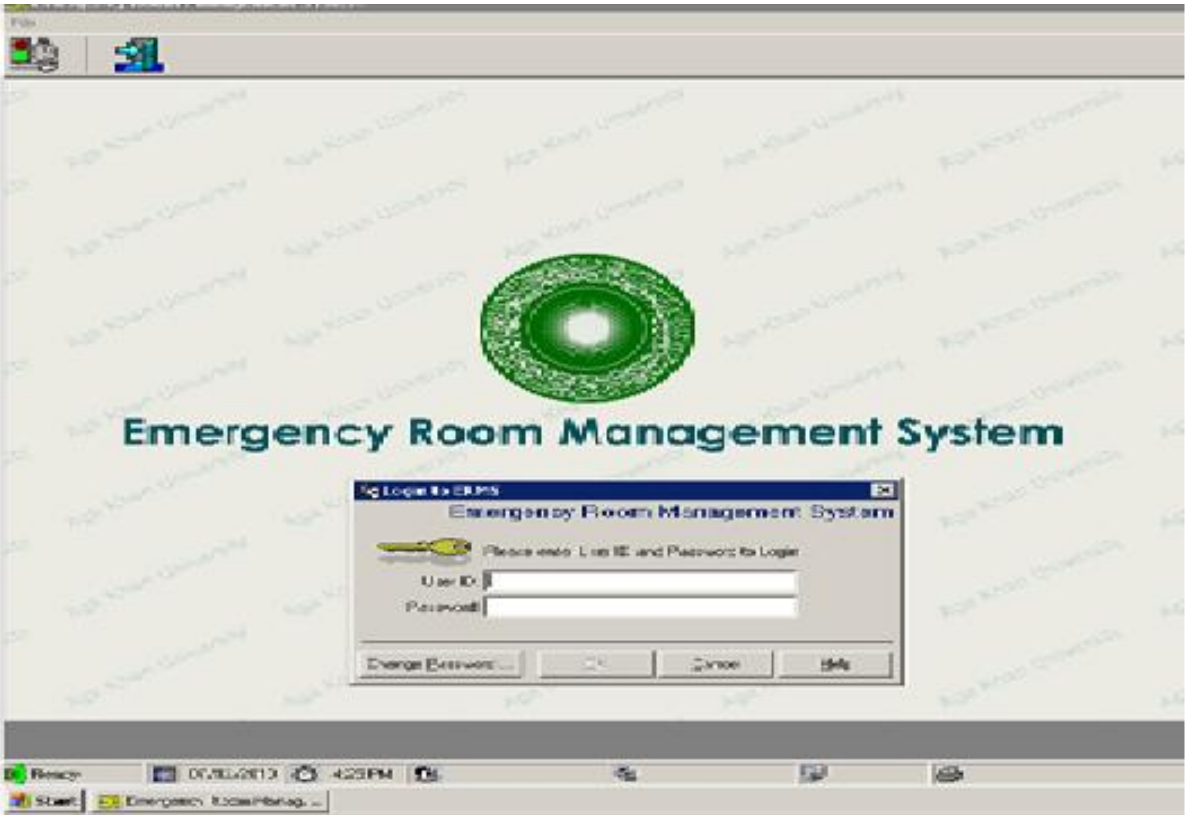
File
Emergency Room Management System
Login to ERMS
Emergency Room Management System
User ID:
Password:
Cancel
Help
Ready
Start

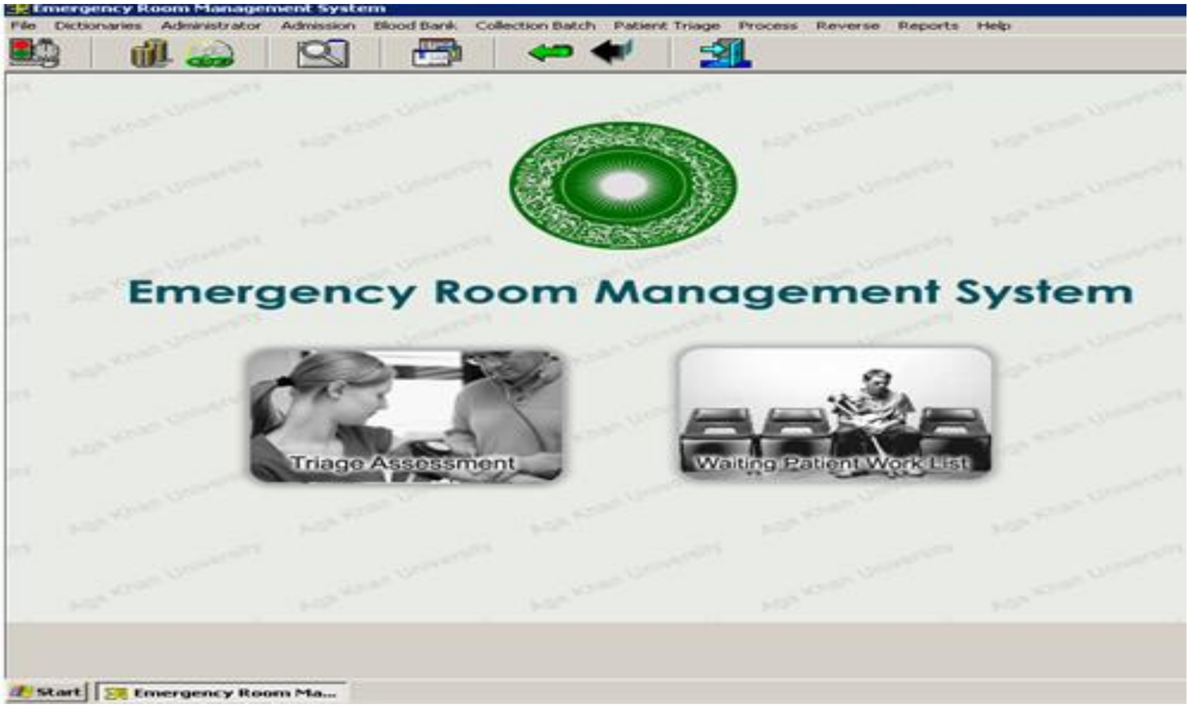
Emergency Room Management System
File Dictionaries Administrator Admission Blood Bank Collection Batch Patient Triage Process Reverse Reports Help
Emergency Room Management System
Triage Assessment
Waiting Patient Work List
Start
Emergency Room Ma...

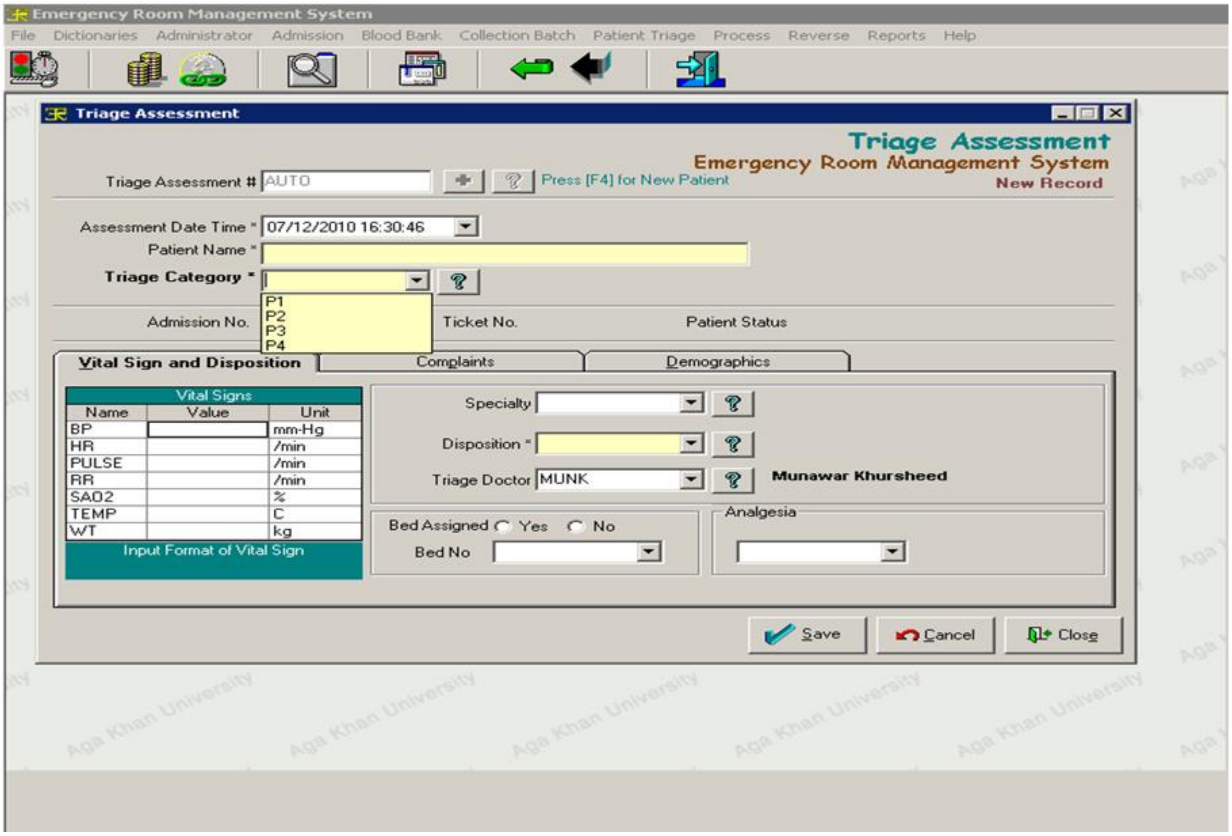
Emergency Room Management System
File Dictionaries Administrator Admission Blood Bank Collection Batch Patient Triage Process Reverse Reports Help
Triage Assessment
Triage Assessment
Emergency Room Management System
New Record
Triage Assessment # AUTO
Press [F4] for New Patient
Assessment Date Time * 07/12/2010 16:30:46
Patient Name *
Triage Category *
P1
P2
P3
P4
Admission No.
Ticket No.
Patient Status
Vital Sign and Disposition
Complaints
Demographics
Vital Signs
Name Value Unit
BP mm-Hg
HR /min
PULSE /min
RR /min
SAO2 %
TEMP C
WT kg
Input Format of Vital Sign
Specialty
Disposition *
Triage Doctor MUNK
Munawar Khursheed
Bed Assigned Yes No
Bed No
Analgesia
Save
Cancel
Close
Aga Khan University

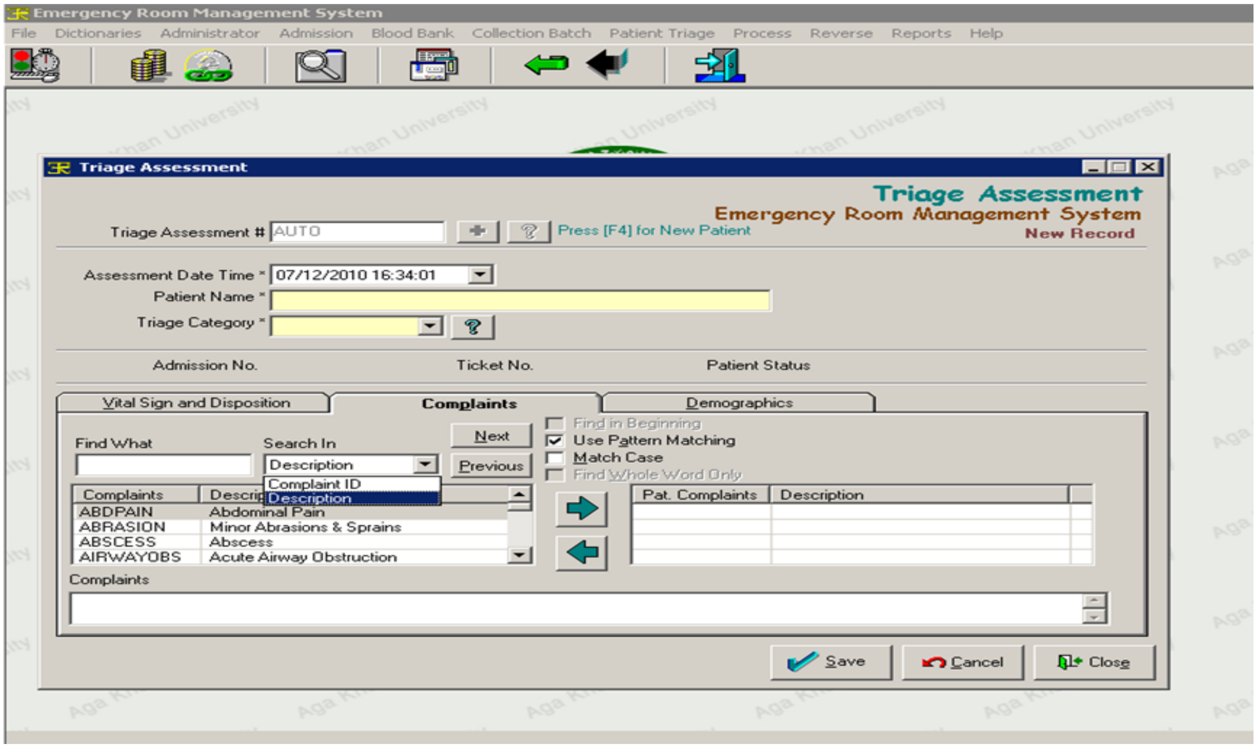
Emergency Room Management System
File Dictionaries Administrator Admission Blood Bank Collection Batch Patient Triage Process Reverse Reports Help
Triage Assessment
Triage Assessment
Emergency Room Management System
New Record
Triage Assessment # AUTO
Press [F4] for New Patient
Assessment Date Time * 07/12/2010 16:34:01
Patient Name *
Triage Category *
Admission No.
Ticket No.
Patient Status
Vital Sign and Disposition
Complaints
Demographics
Find What
Search In
Description
Complaint ID
Description
Next
Previous
Find in Beginning
Use Pattern Matching
Match Case
Find Whole Word Only
Complaints Descrip
ABDPAIN Abdominal Pain
ABRASION Minor Abrasions & Sprains
ABSCESS Abscess
AIRWAYOBS Acute Airway Obstruction
Pat. Complaints Description
Complaints
Save
Cancel
Close
Aga Khan University

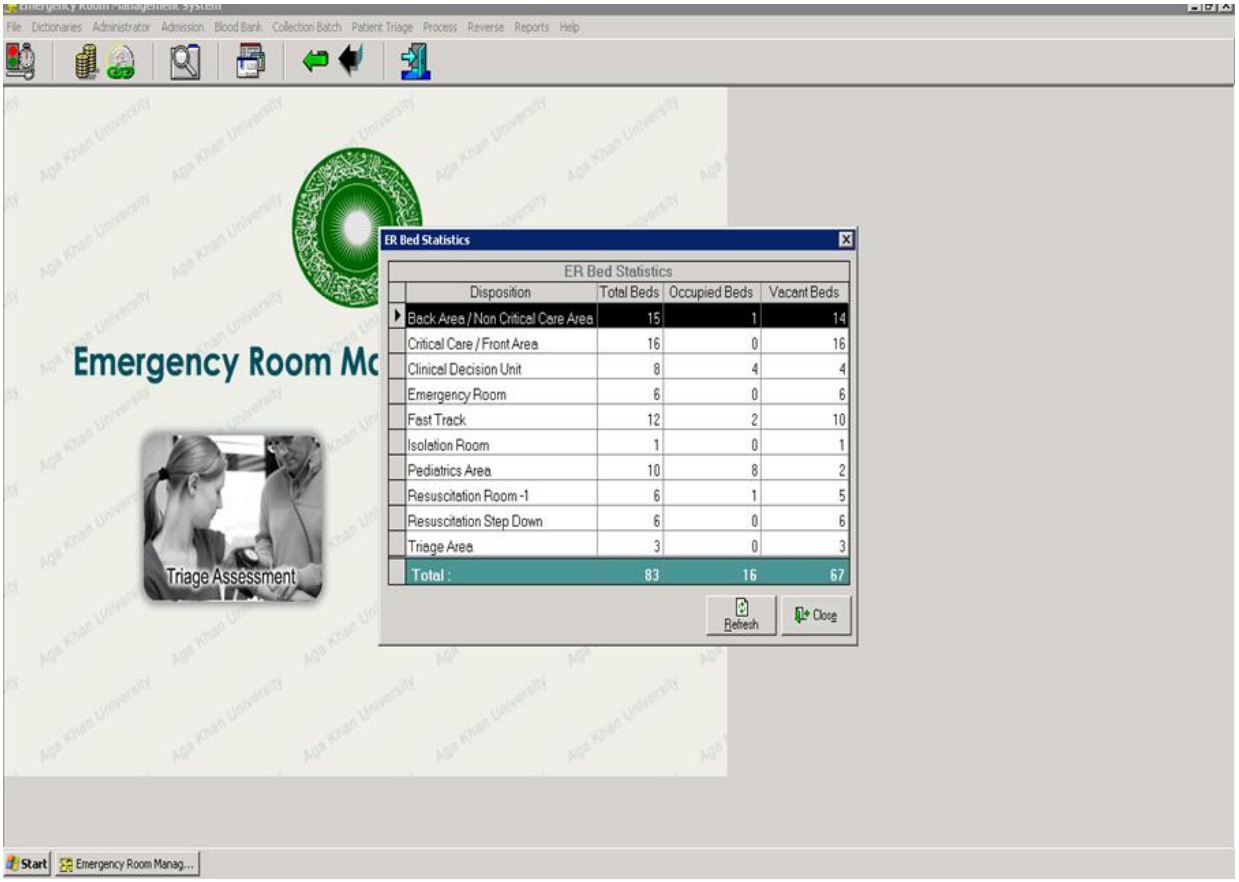

ER Bed Statistics

Disposition	Total Beds	Occupied Beds	Vacant Beds
Back Area / Non Critical Care Area	15	1	14
Critical Care / Front Area	16	0	16
Clinical Decision Unit	8	4	4
Emergency Room	6	0	6
Fast Track	12	2	10
Isolation Room	1	0	1
Pediatrics Area	10	8	2
Resuscitation Room -1	6	1	5
Resuscitation Step Down	6	0	6
Triage Area	3	0	3
Total :	83	16	67
